# General Observations on the Means of Preserving Health in an Army

**Published:** 1839-01-19

**Authors:** Hugh Williamson

**Affiliations:** Surgeon General of the Continental Army


*General Observations on the Means of Preserving Health in an Army, by the late* Hugh Williamson, M. D., L. L. D., *Surgeon General of the Continental Army, in a letter to the late* David Hosack, M. D., L. L. D., F. R. S.

[Communicated to the Editors by Rev. H. W. Ducachet, D.D.]

In the summer season, the troops in general, especially the militia, were employed in active service. They did little garrison duty. In that case they did not suffer much by sickness. Their chief complaints were bilious intermittents. Those fevers, however, were greatly promoted, especially in the Southern states, in the warm season, by the imprudent indulgence of individuals. The writer recollects a particular family, consisting of twelve or thirteen persons, of whom, about the middle of August, there was only one person who was not more or less afflicted with fever. The troops usually finished their day's march by eleven o'clock, A. M., and then, the officers in general, as well as the privates, counted it a luxury, under the shade of a tree, to scrape away the dead leaves, and spreading a blanket on the cool ground, enjoy the pleasure of a siesta. There was, in that family, one person only who never slept nor rested on or near the bare ground. He conceived that the moisture which was commonly ascending from the earth, partly composed of decaying vegetables, must be injurious, therefore he usually reposed on camp stools, or on a plank, or on a large piece of bark taken from a dry tree. That officer alone escaped the fever. Hence it is inferred that troops should be lodged as clear as possible from the moist ground.

There is much reason to believe that two-thirds of the complaints by which military men suffer, originate in the circumstance of sleeping too near the moist ground, by which the perspiration is checked, or moisture is absorbed, that, in most cases, is charged with putrescent vegetable substances.

*Of Troops Encamped.*

From the month of October, 1780, a corps of militia were encamped on the southern border of Virginia, eight or nine months. Their number varied from five to seven hundred. The gentleman who was at the head of the medical staff, resolved, with the approbation of the general officer who had the command, to try how far attention and care would succeed in preserving the health of troops, especially in the winter. The troops were encamped in the midst of a settlement that was entirely composed of small farmers. The position of the enemy indicated that particular post for the camp; and, fortunately, vegetables abounded among these farmers. Besides potatoes of both kinds, and collards, these people, in general, had a small field of turnips, which they never pulled during the winter, except as they wished to eat them. It happened, also, that slabs, planks, or boards were to be obtained with much ease in that vicinity. Availing himself of these circumstances, the medical gentleman referred to, caused the troops to prepare for themselves huts that defended them perfectly from snow or rain. They slept, in general, upon straw, but, in all cases, they were directed to have slabs or boards under the straw, for the ground, except in the middle of winter, was not frozen.

Each mess, of eight or ten, was required to send out a man every day, when the weather was not inclement, in search of potatoes, collards, turnips, onions, or vegetables of some kind. They were well provided with iron pots, or iron camp kettles, and they were required to boil their meat instead of broiling or roasting it. Thus it was that in most cases they had a comfortable dish of soup for dinner, and if there was a lack of vegetables they added the Indian meal to the liquor in which their fresh meat was boiled.

Although vegetables and soup are by no means so nourishing, pound for pound, as flesh, yet it is clear that a more bulky meal, containing more phlegm or watery particles, tend much more than dry flesh to preserve health and prevent colds and fever, by passing off by the pores of the skin. However that may be, the fact is, that, during the winter, spring, and part of the summer, they did not bury more than two men, and none were sent home as invalids.

To obviate the scandal under which some medical gentlemen had laboured, near the main army, being charged with drinking the wine that had been purchased for the sick, care was taken that no wine should be brought into the hospital attached to that camp.

In general, the doctor who presided in that camp, believed that it was not sufficient to give soldiers proper instructions in the mode of conducting themselves, so as to preserve health. He knew that all such bodies of men are apt to be careless. They do not think of probable consequences. He knew also that regimental surgeons and their mates seldom took an interest in the health of the troops. A sickly army was counted a general calamity. They did not trouble themselves in attempting to prevent such calamity. He had known too many instances of their criminal indolence, neglect, and dissipation. They pampered themselves at the expense of the sick. Instances he had known of a regimental surgeon selling wine and sugar to officers of his regiment, which had been put into his hands for the benefit of the sick, but that very surgeon paid very little attention to the sick. The more of them who died, the proofs were the stronger of a sickly camp.

Being possessed of such information, the gentleman who had the care of the camp, above mentioned, did not count it sufficient to give proper directions to regimental surgeons, or their assistants; he visited the several huts in person every second or third day, to see whether his orders were duly executed. And the neglect or bad conduct of any surgeon was sure to be followed by a discharge from the service.

In a word, it was his fixed opinion that one-third of the deaths that happen in camps, may be traced to the ignorance or criminal neglect of the medical gentlemen attached to the army.

